# First detailed karyo-morphological analysis and molecular cytological study of leafy cardoon and globe artichoke, two multi-use Asteraceae crops

**DOI:** 10.3897/CompCytogen.v10i3.9469

**Published:** 2016-09-26

**Authors:** Debora Giorgi, Gianmarco Pandozy, Anna Farina, Valentina Grosso, Sergio Lucretti, Andrea Gennaro, Paola Crinò, Francesco Saccardo

**Affiliations:** 1 ENEA R. C. Casaccia, Italian National Agency for New Technologies, Biotechnologies and Agro-industry Division, Via Anguillarese 301, 00123 Roma, Italy; 2 Tuscia University, Department of Agriculture, Forests, Nature and Energy (DAFNE), Via S.C. de Lellis, 01100 Viterbo, Italy; 3 European Food Safety Authority, GMO Unit, Via Carlo Magno 1A 43126 Parma, Italy

**Keywords:** Cynara, SSR simple sequence repeats, repetitive sequences, flow cytometry, FISHIS, FISH

## Abstract

Traditionally globe artichoke and leafy cardoon have been cultivated for use as vegetables but these crops are now finding multiple new roles in applications ranging from paper production to cheese preparation and biofuel use, with interest in their functional food potential. So far, their chromosome complements have been poorly investigated and a well-defined karyotype was not available. In this paper, a detailed karyo-morphological analysis and molecular cytogenetic studies were conducted on globe artichoke (Cynara cardunculus Linnaeus, 1753 var. scolymus Fiori, 1904) and leafy cardoon (Cynara cardunculus Linneaus, 1753 var. altilis De Candolle, 1838). Fluorescent *In Situ* Hybridization In Suspension (FISHIS) was applied to nuclei suspensions as a fast method for screening of labelling probes, before metaphase spread hybridization. Classic Fluorescent *In Situ* Hybridization (FISH) on slide, using repetitive telomeric and ribosomal sequences and Simple Sequence Repeats (SSRs) oligonucleotide as probes, identified homologous chromosome relationships and allowed development of molecular karyotypes for both varieties. The close phylogenetic relationship between globe artichoke and cardoon was supported by the very similar karyotypes but clear chromosomal structural variation was detected. In the light of the recent release of the globe artichoke genome sequencing, these results are relevant for future anchoring of the pseudomolecule sequence assemblies to specific chromosomes. In addition, the DNA content of the two crops has been determined by flow cytometry and a fast method for standard FISH on slide and methodological improvements for nuclei isolation are described.

Fluorescent *In Situ* Hybridization In Suspension

Fluorescent *In Situ* Hybridization

Simple Sequence Repeats

## Introduction

The globe artichoke (Cynara cardunculus Linnaeus, 1753 var. scolymus (L.) Fiori, 1904) and the cultivated leafy cardoon (Cynara cardunculus Linneaus, 1753 var. altilis De Candolle, 1838) are dicotyledonous angiosperms belonging to the family *Asteraceae* and originate from the Mediterranean area (Sonnante et al. 2007a, b). They contribute significantly to the agricultural economy of this area, mainly of Italy, Egypt, Spain, France, Algeria and Morocco, which yields more than 70% of the total world globe artichoke production of 1.70 Mtons (FAOSTAT 2013). Peru, Argentina, China and USA are emerging countries for artichoke production outside Mediterranean region.

In spite of the agronomic, nutritional and industrial importance of globe artichoke and leafy cardoon for the Mediterranean basin, their genetics and cytogenetics is relatively poorly characterized, as recently stated by Scaglione et al. (2016). The unambiguous identification of individual chromosomes in the karyotype of a species is a cornerstone in understanding the genome organization and in identifying useful genes for breeding, but the small size and the remarkable similarity in the chromosome morphology (Falistocco 2016) still represent a challenge in defining a detailed karyotype for both *Cynara* varieties.

In addition to standard chromosome morphological analysis, cytogenetics can take advantage of a molecular approach based on fluorescence *in situ* hybridization (FISH) of repetitive sequences on metaphase chromosomes. This approach is very informative in recognising individual chromosomes and in delineating the structure and composition of genomic regions (Jiang and Gill 2006; Chester et al. 2010). This methodology enables the physical localization of one or more DNA probes along chromosomes. Among the different classes of repetitive sequences, SSRs represent one of the most valuable cytological markers in chromosome discrimination (Sharma et al. 2007; Cuadrado et al. 2008) due to their abundance and wide distribution in plant genomes (Heslop-Harrison and Schwarzacher 2011). In addition, the repeat sequences coding for ribosomal DNA (rDNA) have been widely used to characterize plant chromosome complements (Jiang and Gill 1994; Sharma et al. 2012). In the present study, a detailed karyo-morphological analysis and FISH characterization using a number of probes, that is, SSR derived oligonucleotides, telomeric repeats and the 18S-5.8S-26S rDNA, were performed to produce the first measures of single chromosomes and the molecular cytogenetic characterization of the globe artichoke and cardoon complements. FISHIS (Giorgi et al. 2013a) was used on nuclei suspensions as a fast and effective way to screen and select probes producing strong and localized signals, particularly useful in those species, such as *Cynara cardunculus*, where mitotic index remained quite low, even after using mitosis blocking agents (Giorgi et al. 2013b). Finally, flow cytometry genome size estimation was performed for both crops.

## Materials and methods

### Plant materials

Globe artichoke cultivar (cv) Istar and cardoon cv Bianco Avorio seeds were kindly provided by the Seed Company Topseed (Sarno, Salerno, Italy) while *Pisum sativum* (Linnaeus, 1573) cv Citrad seeds were generously provided by Dr. J. Doležel (Centre of Plant Structural and Functional Genomics, Institute of Experimental Botany, Olomouc, Ceck Republic). For both DNA content determination and cytogenetic analysis, *Cynara* spp. seeds were germinated in the dark on moist filter paper at 24±1 °C for 5-10 days, after a hot treatment at 50 °C for 10 min (for *Pisum sativum*, no hot treatment was performed). Actively growing roots and young leaves were excised for further treatment.

### Nuclei isolation and DNA staining

Nuclei were extracted from 50 mg of young leaves using two different protocols. The first was performed according to Doležel and Greilhuber (2010) using fresh tissue while the second was carried out on tissue fixed in 2% formaldehyde solution/Tris buffer (10 mM Tris, 10 mM Na_2_EDTA.2H_2_O, 100 mM NaCl and 0.1% Triton X-100) for 20 min at 4±0.5 °C. After rinsing in Tris buffer, three times for 5 min each at 4 ±0.5 °C, leaves were briefly chopped in 1 ml of lysis buffer LB (15 mM Tris, 2 mM Na_2_EDTA, 0.5 mM sperminetetrahydrochloride, 80 mM KCl, 20 mM NaCl, 15 mM β-mercaptoethanol, 0.7% (v/v) Triton X-100; pH 7.5) and homogenized with Ultraturrax T10 and G5 generator (IKA, Germany) at 10.000 rpm for 5 sec. The resulting homogenate was filtered through a 36 µm nylon mesh to remove debris, and the nuclei stained with 75 µM propidium iodide (PI) and 100 µg ml^-1^ RNase (Doležel et al. 1989).

### Genome size determination

Flow cytometric estimation of nuclear DNA content stained with PI (λ ext max: 293 nm and 514 nm; λ ems max 625 nm) was performed using a FACS Vantage SE flow cytometer (BD Bioscience, San Jose, CA) with a solid state laser (Genesis CX STM, Coherent, Santa Clara, CA), UV emission at 200 mW, and a 70 µm flow tip running at 27 psi with a solution of 50 mM NaCl as sheath fluid. *Pisum sativum* cv “Citrad” was used as internal standard (2C = 9.09 pg) (Doležel et al. 1998). Nuclei of globe artichoke, cardoon and pea were isolated, stained and simultaneously analysed. The histogram of DNA fluorescence intensity was obtained following flow cytometric analysis of PI-stained nuclei. The flow cytometer was set for measuring with a CV of 2.5% or better; the measurements of relative florescence intensity of stained nuclei were performed on a linear scale, and typically 5.000–10.000 nuclei were analysed for each sample (samples were run in triplicates). The peak of fluorescence of G0/G_1_ nuclei from *Pisum sativum* was tuned to mean channel 400. The genome size (pg DNA) of globe artichoke and cardoon was calculated using DNA fluorescence measurements and the following equation:

unknown 2C DNA content = [(unknown G_1_ peak mean)/ (standard G_1_ peak mean)] × standard 2C DNA content.

2C DNA content (pg) was converted to base pairs (bp) following the factor: 1 pg DNA = 0.978 × 10^9^ bp (Doležel et al. 2003).

### Chromosome slide preparation and DNA staining

Actively growing roots were excised and pre-treated in 2 mM 8-Hydroxyquinoline (8HQ) for 3-5 h at room temperature (r.t.) or in 30 µM Oryzalin for 20 h at 4 °C, and then fixed in Carnoy solution (ethanol : glacial acetic acid = 3:1) at -20 °C for at least 18 h. Chromosome spreads were prepared according to the “drop spreading method” developed by Andras et al. (1999). Chromosomes were stained for morphometric analysis and karyotype definition with 4,6-diamidino-2-phenylindole (DAPI) at a final concentration of 2 µg ml^-1^, according to Schwarzacher and Heslop-Harrison (2000).

### FISH

In order to better discriminate each chromosome and pairing homologous chromosomes, single and double-target fluorescence *in situ* hybridization (FISH) was performed using ribosomal DNA (rDNA) sequences, telomeric and synthetic SSR oligonucleotides.

The 18S-5.8S-26S rDNA clone pTa71 (Gerlach and Bedbrook 1979) was labelled by nick-translation with Cy3 using standard kits (Nick Translation Mix, Roche) according to the manufacturer’s instructions. FISH with the pTa71 probe was performed according to Andras et al. (1999) with minor modifications: the hybridization mixture (40 µL/slide) was prepared by adding 50% (v/v) formamide, 10% (w/v) dextran sulphate, 10% (v/v) 20X SSC and 120-160 ng of probe per slide, denatured at 75 °C for 10 min, and then put on ice.

Metaphases were denatured at r.t for 5 min in 70% ethanol (pH13 using 4 N NaOH). Preparations were dehydrated at r.t. through an ice-cold ethanol series (70%, 85% and 100%) for 2 min each, and air dried. The denatured probe was applied after chromosomes alkaline denaturation and plastic cover slips were placed over the specimens and the slides were incubated in a humid chamber at 37 °C for 16 h. After hybridization, the coverslips were removed and the preparations were subjected to a single stringency wash in 50% (v/v) formamide in 1 x SSC, followed by 2 additional washes in 2 x SSC, for 5 min at 45 °C each. Finally, samples were counterstained with DAPI and mounted in a Vectashield antifade solution (Vector Laboratories, Burlingame, CA, USA).

### Fluorescence *In Situ* Hybridization In Suspension (FISHIS)

Before FISH analysis on metaphase spreads, a selection of SSR oligonucleotides was carried out on nuclei using FISHIS. Nuclei were isolated from roots following the same procedure previously described for fixed leaves. Hybridization was performed according to Giorgi et al. (2013a) using these SSR-oligonucleotides as probes: (AT)_12_, (AAC)_5_, (AGG)_5_, (ATC)_5_, (GAA)_7_, (CAT)_5_, (CA)_10_, (GA)_10_, (CAG)_5_, (GACA)_4_, (TTTAGGG)_5_ and only those with clear hybridization signals were selected for FISH on slide. The SSR probes were single stranded oligos labeled at one end with FITC (fluorescein-5-isothiocyanate) or Cy3 (Cyanine 3), synthesized by Eurofins MWG Operon (Ebersberg, Germany). After FISHIS, 3–5 µl of labelled nuclei suspension was placed on a glass slide and mounted in Vectashield antifade solution (Vector Laboratories, Burlingame, CA, USA) for microscope image analysis.

### Fast FISH

A fast FISH method was developed and carried out on metaphase spreads of artichoke and cardoon using selected SSR oligonucleotides as probes. Chromosome DNA was denatured in an alkaline 70% ethanol solution, as previously described, and the preparations were dehydrated at r.t. through an ice-cold ethanol series (70%, 85% and 100%) for 2 min each, and air dried. A mix containing 1.5 - 3 ng µl^-1^ of labelled oligonucleotide in 2X SSC (300 mM sodium chloride, 0.3 mM trisodium citrate) was applied on the slide (final volume 60 µl per slide) and hybridization was carried on at r.t. for 1 h After washing in 4X SSC, 0.2% Tween20 for 10 min, samples were counterstained with DAPI and mounted in antifade solution.

### Microscope and image analysis

Microscope slides with chromosomes or nuclei were examined with a Nikon Eclipse TE2000-S inverted microscope equipped with an HB0100 W lamp and a CFI Plan Apo oil objective 100X and appropriate filter sets for DAPI, FITC and Cy3 fluorescence. Separate images from each filter set were digitalized and analysed using a DXM1200F Nikon camera and the NIS AR 3.1 software (Nikon Instruments S.p.A, Florence, Italy), respectively. Image analysis and measurements were performed using ImageJ v1.46 (Abramoff et al. 2004). Chromosomes were arranged in decreasing order according to their total length (µm) using the ImageJ plugin Chias IV. Manual adjustment and chromosome pairing were performed also according to FISH hybridization results. Chromosomes were classified on the basis of arm ratio (AR = length of the long arm/length of the short arm) as metacentric (M, AR = 1.00÷1.49), submetacentric (SM, AR = 1.50÷2.99) or acrocentric (A, AR > or = 3), following Guerra’s nomenclature (Guerra 1986).

## Results

### Flow cytometric analysis of DNA content

**Figure 1.**
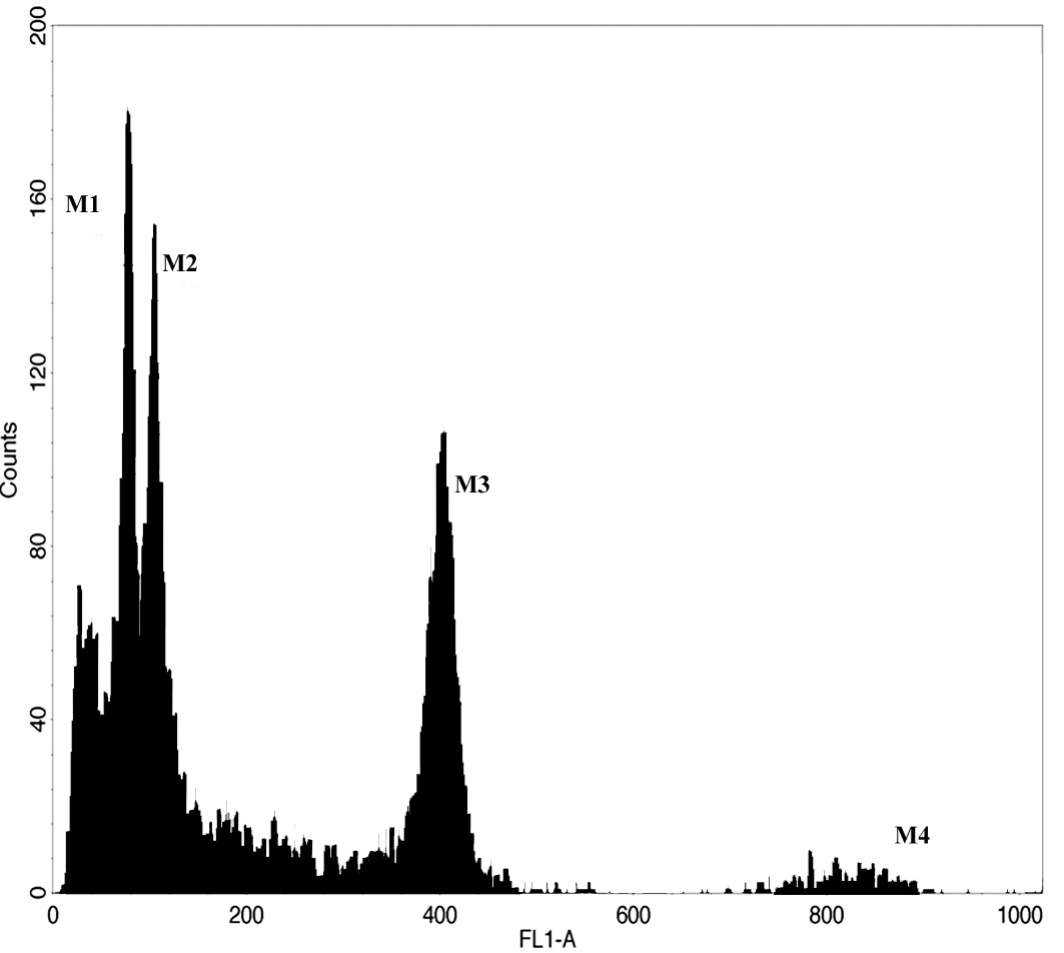
Flow Cytometry DNA content histogram. Flow cytometry analysis of DNA fluorescence peaks (FL1A) from G0/G_1_ (2C) PI stained leaf nuclei from cultivated cardoon (M1), globe artichoke (M2) and *Pisum sativum* (pea) (M3). Pea was used as an internal standard and the peak was set at channel 400; M4 shows pea G_2_ nuclei (4C).

After optimization of the isolation procedure, flow cytometry estimation of nuclear DNA content was performed analysing nuclei isolated from globe artichoke and cardoon using *Pisum sativum* cv. Citrad as internal standard, When using the most common isolation buffers and the classical method of chopping of fresh tissues (Doležel and Greilhuber 2010), a low yield for *Cynara* nuclei suspensions was obtained. An increased nuclei yield (of about 5 times) was achieved after fixation of fresh tissues in 2% formaldehyde, standard chopping of leaves in LB isolation buffer, containing a larger amount of Triton-X100, and by performing an additional homogenization step. These modifications resulted in an average amount of 600 nuclei per milligram of plant tissue, sufficient for an operational flow cytometric analysis, and were effective to get a good DNA fluorescence histogram, with a low noise background (Figure 1). The DNA content for cardoon and globe artichoke was estimated to be 2C = 2.20 pg ± 0.04 and 2C = 2.40 pg ± 0.04 (Figure 1) corresponding to a C genome size of 1.07 ×10^9^ bp and 1.17 ×10^9^ bp, respectively.

### Cytogenetics: morphometric analysis

In order to perform a good morphological analysis, the quantity and quality of metaphase spreads, in terms of absence of cytoplasm and low percentages of overlapping chromosomes, is of critical relevance. Here a pre-treatment with oryzalin, as antimitotic agent, was tested for the first time in *Cynara* and compared to the 8-hydroxyquinoline used in previous studies; a further slight increase in methaphases number (about 5%) was observed.

**Figure 2.**
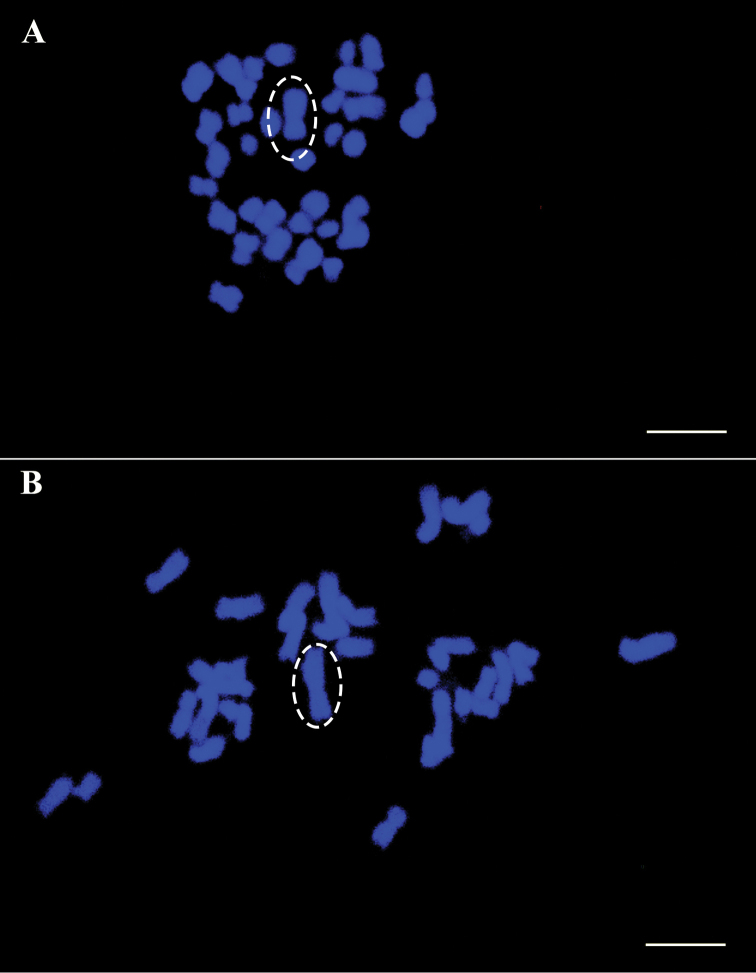
The smallness and variable sizing of *Cynara* chromosomes is shown; in both panel **A** and **B**, leafy cardoon chromosome 1 has been circled as an example of the different condensation level of the same metaphase chromosomes. In **A** chromosome 1 is 3.2 µm, in **B** it is 4.3 µm (a 36% size variation). Scale bars: 5 µm.

**Figure 3.**
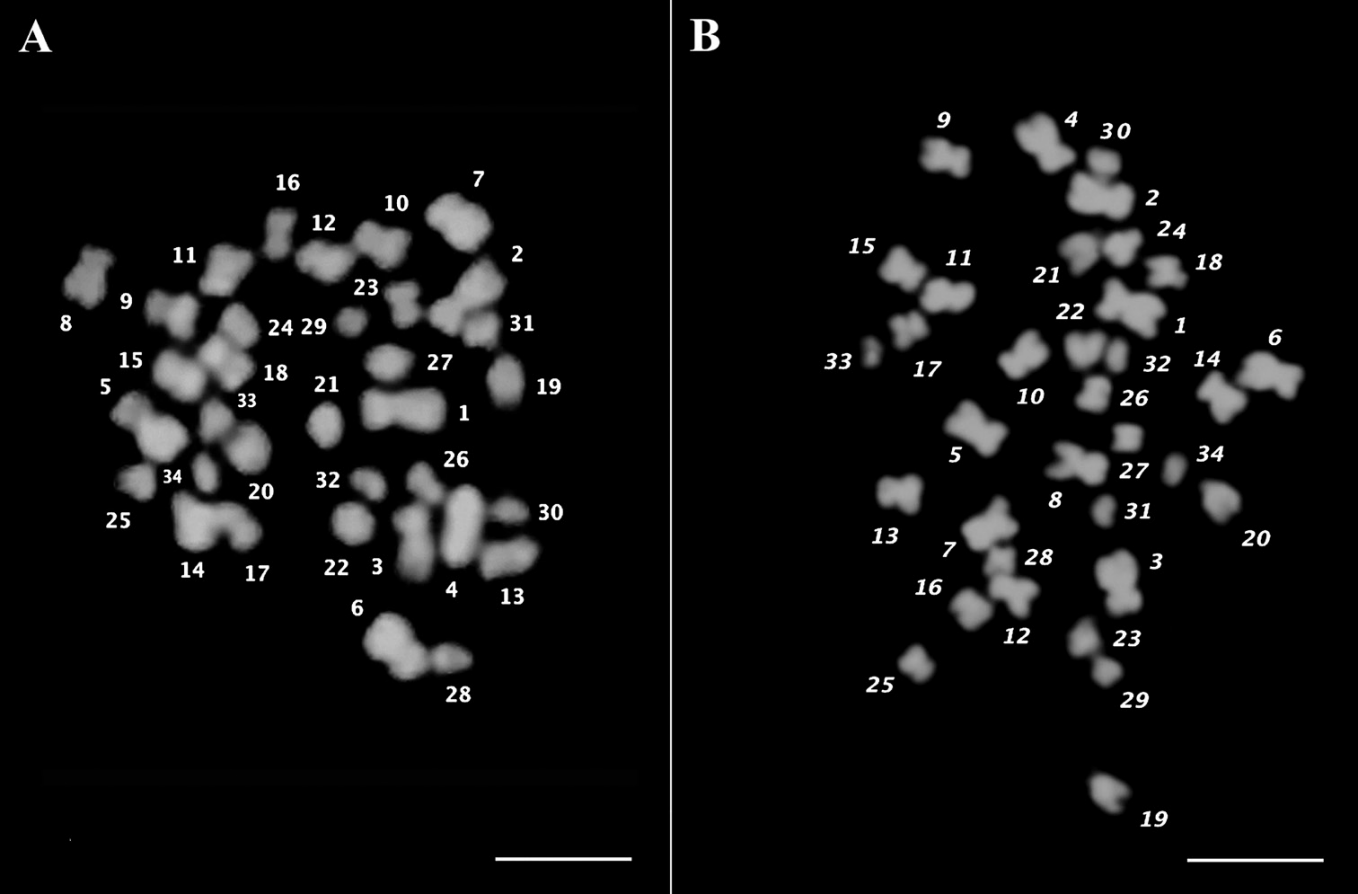
Chromosome numbering. Metaphase spreads showing numbered chromosomes of cardoon (**A**) and globe artichoke (**B**) counterstained with DAPI. Scales bars: 5 µm.

The morphometric analysis of cardoon and globe artichoke chromosomes was carried out by measuring the total length (tl), the arm length and the arm ratio of all 34 individual chromosomes (Table 1, A and B, respectively). Based on their size, chromosome pairs of both varieties could be divided into three main groups: large (2.1µm<tl<3.2µm), medium (1.6µm<tl<2.0µm) and small (tl<1.6µm), with seven, six and four chromosome pairs, respectively. Within each group, chromosomes were similar in size and morphology and difficult to distinguish and arrange. Large differences in chromatin condensation made it difficult to compare measurements from different metaphases in the same preparation (Figure 2A and 2B). Figure 3 shows two typical mitotic metaphase spreads where chromosome sizes are close in the two crops, ranging from 3.19–3.16 µm for the biggest chromosome pairs, to 1.22 µm for the smallest ones in globe artichoke and cardoon, respectively. The calculated karyotypic formula is similar for the two crops, 2n = 16M+8SM+10A for Cynara cardunculus L. var. scolymus (L.) Fiori and 2n = 16M+6SM+12A for Cynara cardunculus L. var. altilis DC, the only exception being chromosome pair 14, which is acrocentric in the cultivated cardoon and metacentric in globe artichoke, as shown at the ideogram.

**Table 1.** Morphometric analysis of *Cynara cardunculus* chromosomes. Morphological analysis of the 2n = 34 chromosomes of cultivated cardoon (**A**) and globe artichoke (**B**) based on Fig. 3A, B, respectively. Abbreviations: **C.p.** Chromosome pairs **T.l.** Total length **p** Average length of short arm **q** Average length of long arm; **AR** Arm Ratio (p/q) **Class** classification: **m** metacentric **sm** submetacentric **a** acrocentric.

C.p.	T.l. (µm)		p (µm)		q (µm)		AR		Class	
	A	B	A	B	A	B	A	B	A	B
1	3.16	3.19	1.23	1.27	1.93	1.92	1.57	1.51	s	s
2	2.97	3.16	1.07	1.24	1.78	1.93	1.78	1.55	sm	sm
3	2.58	2.81	1.05	1.21	1.55	1.60	1.46	1.32	m	m
4	2.35	2.65	0.97	1.16	1.38	1.49	1.42	1.28	m	m
5	2.35	2.45	0.78	0.95	1.57	1.50	2.01	1.57	sm	sm
6	2.12	2.27	0.85	0.95	1.27	1.32	1.49	1.39	m	m
7	2.10	2.25	0.87	0.93	1.23	1.32	1.41	1.42	m	m
8	2.03	2.00	0.79	0.78	1.24	1.22	1.56	1.56	sm	sm
9	2.00	1.98	0.92	0.92	1.08	1.06	1.17	1.15	m	m
10	2.00	1.98	–	–	2.00	1.98	>3	>3	a	a
11	1.95	1.95	–	–	1.95	1.95	>3	>3	a	a
12	1.79	1.95	0.81	0.71	0.98	1.24	1.20	1.74	m	sm
13	1.68	1.70	0.62	0.79	1.06	0.91	1.70	1.15	sm	m
14	1.40	1.41	–	0.65	1.40	0.76	>3	1.17	a	m
15	1.30	1.38	–	–	1.30	1.38	>3	>3	a	a
16	1.25	1.27	–	–	1.25	1.27	>3	>3	a	a
17	1.22	1.22	–	–	1.22	1.22	>3	>3	a	a

### Molecular cytogenetics: rDNA localization FISHIS and FISH with SSR-oligonucleotides

**Figure 4.**
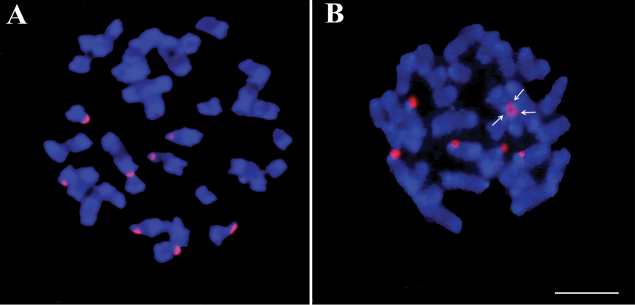
FISH molecular cytogenetic analysis with rDNA. FISH on metaphase spreads of cultivated cardoon (**A**) and globe artichoke (**B**) using the rDNA probe pTa71-Cy3 (red fluorescence). In Fig. 3B, arrows point to the hybridization spots localized on the three small acrocentric chromosomes, placed head to head. Scale bar: 5 µm.

FISH localization of rDNA was investigated using the pTa71 sequence as a probe. For both crops there were eight hybridization signals (Figures 4A and 4B), localized at the very distal part of chromosomes, and sometimes appearing almost detached from them (data not shown). Detailed morphological analysis identified very small satellite bodies on some acrocentric chromosomes, which were not consistently present, probably because they were damaged during slide preparation and FISH labelling. However, according to our observations, we identify two medium acrocentric and two small acrocentric chromosomes (most probably chromosomes 15 and 16) as the ones having satellites.

**Figure 5.**
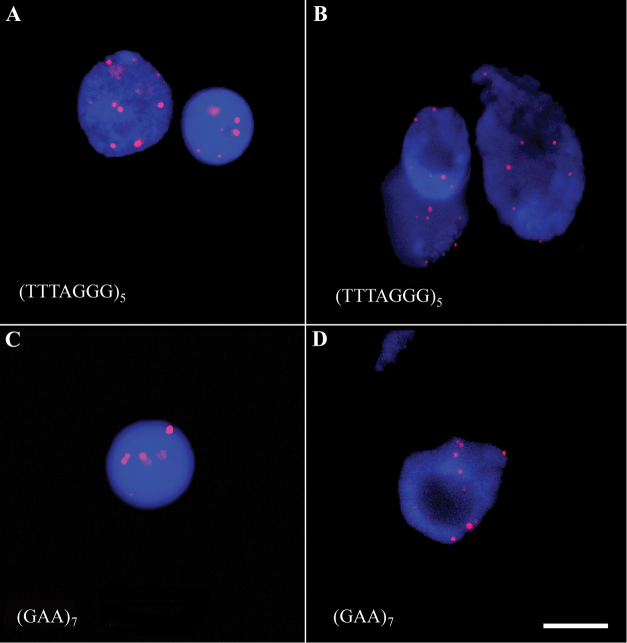
Fast screening of labelling probes was performed by FISHIS on cultivated cardoon (panel **A** and **C**) and globe artichoke (panel **B** and **D**) nuclei suspensions. The nuclei with the clearest and discrete telomeric (TTTAGGG)_5_ and SSR (GAA)_7_ signals are shown. All oligonucleotides were fluorescently labelled by Cy3 (red fluorescence). Nuclear DNA was counterstained with DAPI (blue fluorescence). Scale bar: 5 µm.

Screening SSR by FISHIS analysis revealed that only the telomeric sequence (TTTAGGG)_5_ and the oligonucleotide (GAA)_7_ showed clear and discrete hybridization signals on nuclei of both crops (Figure 5); while the two di-nucleotide (CA)_10_ and (GA)_10_ probes had a weak and diffuse signals (data not shown).

**Figure 6.**
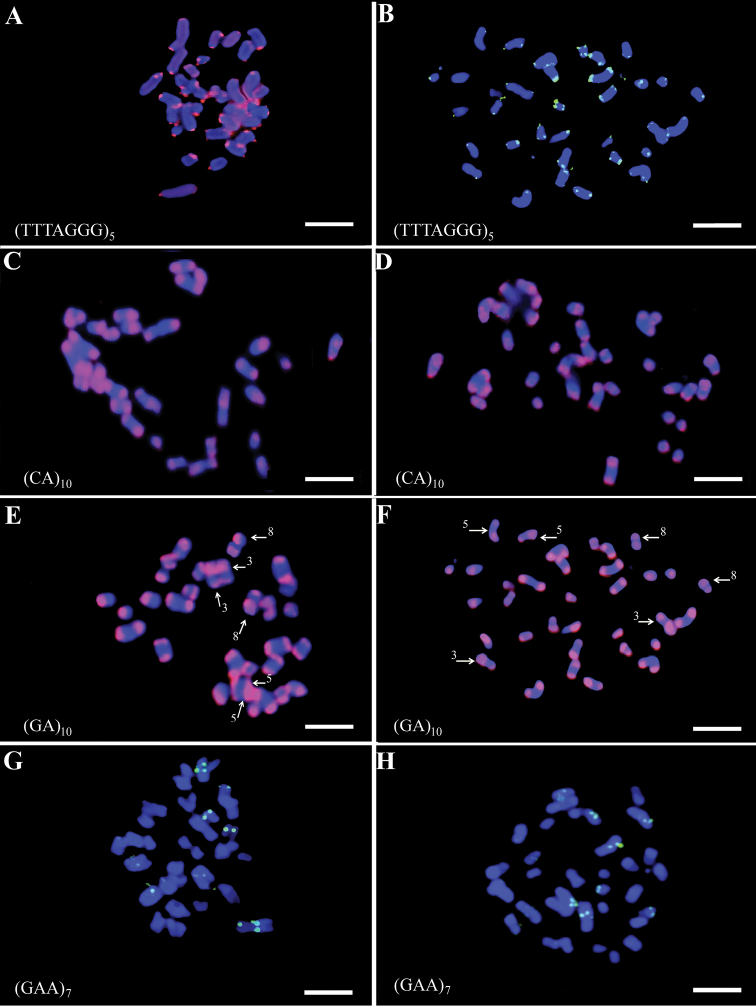
FISH molecular cytogenetic analysis with SSR probes. FISH on metaphase spreads of cardoon (left hand side) and of globe artichoke (right had side). The oligonucleotides sequence is indicated in each panel. Oligonucleotides were labelled with Cy3 (red fluorescence) or FITC (green fluorescence) fluorochromes. In (**E**) and (**F**) chromosomes 3, 5 and 8 of cardoon and globe artichoke, respectively, are indicated by arrows and can be discriminated by the widespread (GA)_10_ hybridization pattern on the long arms. Scale bars: 5 µm.

All four oligonucleotides (TTTAGGG)_5_, (GA)_10_, (CA)_10_, and (GAA)_7_ were used for fast standard FISH on chromosome spreads. As expected, (TTTAGGG)_5_ hybridized at the telomeres, facilitating identification of the ends of the chromosome and more accurate measurements (Figures 6A and B).

**Figure 7.**
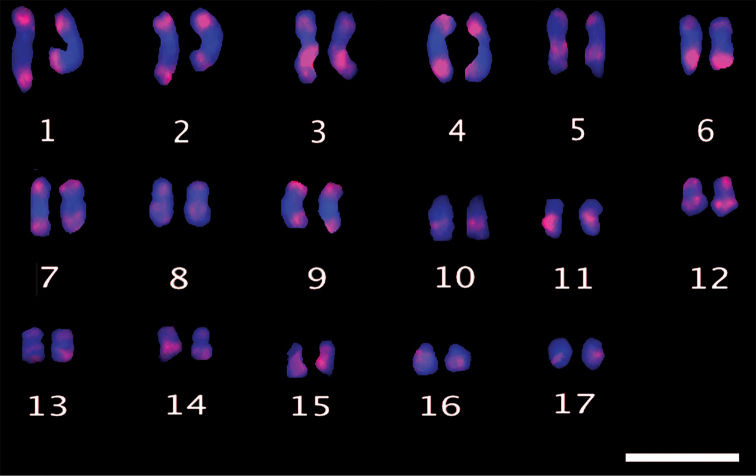
An example of globe artichoke pairing of homologous chromosomes based on (GA)_10_ labelling pattern. Scale bar: 5 µm.

The (CA)_10_ and (GA)_10_ di-nucleotides showed very similar hybridization patterns on the chromosomes of the two crops (Figures 6C-F). In most chromosomes, hybridization signals were localized mainly at the telomeric and subtelomeric regions of both arms, with a slightly different band sizes, which helped in pairing homologous chromosomes (Figure 7). On chromosome pairs 3, 5 and 8, the extent of the hybridization signal was more widespread on the long arm (Figures 6E and 6F). Cardoon and globe artichoke acrocentric chromosomes can be discriminated from all other chromosomes by the distribution of the two SSR oligonucleotides along only on one side of the chromosome arms.

A less clear hybridization pattern was obtained using the (GAA)*_7_* probe which showed a variable and sometimes asymmetric distribution of the signals on sister chromatids, mainly on the large chromosome of globe artichoke and cardoon. For at least two large, one medium and one small chromosome pair a hybridization signal was visible in all the observed metaphases for both crops (Figures 6G and 6H), but further analyses are required.

**Figure 8.**
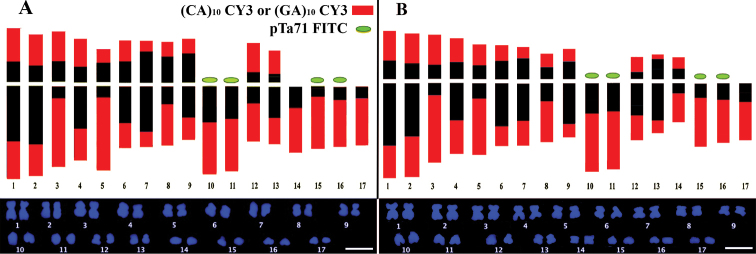
Ideogram with molecular characterization of cardoon and globe artichoke complement. Ideogram showing chromosome morphology (in black) and the (CA)_10_, (GA)_10_ di-nucleotides (red) and pTa71 sequence (green) distribution on cardoon (**A**) and globe artichoke (**B**) complement. The two di-nucleotides localize at similar chromosome regions. Scale bar: 5 µm.

Ideograms summarising chromosome morphology and molecular karyotyping with the (CA)_10_ /(GA)_10_ and pTa71 DNA probes of the two crops are shown in Figure 8.

## Discussion

In spite of the recent release of globe artichoke genome sequence (Scaglione et al 2016), the physical structure in which DNA is organized, that is the chromosomes, has been poorly investigated for cardoon and globe artichoke. The knowledge of chromosome organisation is important for studies of plant species evolution and is also relevant in plant breeding programmes. In the present paper, after the DNA content determination, we developed a detailed karyo-morphological analysis and a FISH-based molecular characterization of the chromosome complement of both crops

The genomes size measured in this study for leafy cardoon (2C = 2.20 pg) and globe artichoke (2C = 2.40 pg) are slightly different to those previously reported by Khaldi et al. (2014) and Marie and Brown (1993), respectively. Khaldi et al. (2014) reported a range of variability in DNA content, from 2C = 1.98 to 3.03 pg, in 10 populations of wild cardoon, and 2C = 2.05 and 2.10-2.11 pg for a single variety of globe artichoke and for two varieties of cultivated cardoon, respectively, while Marie and Brown (1993) stated a DNA content of 2C = 2.22 pg for globe artichoke. The differences between our estimates in DNA content and those of Marie and Brown (1993) and Khaldi et al. (2014) can be attributed to the DNA content estimation for the internal standard (*Pisum sativum*). Both works used 2C = 8.37 pg, while we employed 2C = 9.09 pg from Doležel et al. (1998). Possible intra-specific genome size variability was reported by Khaldi et al. (2014) which may also account for differences in DNA content estimates.

For cytogenetic studies in globe artichoke we have previously tested several microtubule assembling inhibitors, that is, 8-hydroxyquinoline, amiprophos-methyl, colchicine and a-bromonaphtalene, to increase the number of metaphases. 8-hydroxyquinoline was identified as the most effective inhibitor but even so, the mitotic index of *Cynara* remained as low as 10% (Giorgi et al. 2013b). Here a further slight increase in metaphase number was obtained using oryzalin.

To enhance metaphase spread quality, a drop spreading method recommended for plants with small chromosomes was used (Andras et al. 1999). This method produced good results in terms of cytoplasm removal and reduction of overlapping chromosomes, but it required a large number of root apexes to be processed. Considering that root tips are composed of cells at different cell cycle stages we observed high variation in the level of chromatin condensation among metaphases on the same slide, even using antimitotic drugs. This heterogeneous chromosome condensation can make it difficult to clearly identify chromosome structures such as centromere position and secondary constrictions, and complicates chromosome length measurements. The morphological analysis and FISH signals of the chromosome complements of cultivated cardoon and globe artichoke revealed similar karyotypes, with analogous rDNA gene and SSR distribution in the two crops, supporting their close phylogenetic relationship. In fact, Fiori (1904) and Wiklund (1992) considered that both wild cardoon and the cultivated crops (cardoon and globe artichoke) belong to the same species (i.d. *Cynara cardunculus* L). Further studies indicated that wild cardoon is the common progenitor for both crops, which subsequently diverged in type of reproduction system and end use, probably following two distinct domestication events, separated in time and space (Sonnante et al. 2007b; Gatto et al. 2013). Our karyo-morphological analysis of globe artichoke and cardoon is largely different from the basic study reported by Falistocco (2016) in which all chromosomes of both crops were defined as metacentric and divided into three groups without showing single chromosome measurements. In the present study, a number of sub-metacentric and acrocentric chromosomes were identified and a clear difference was observed between the two crops in chromosome 14, which is acrocentric in cardoon and metacentric in artichoke. Such structural chromosome differences could indicate either the deletion of the small arm of the cardoon, or a translocation or insertion on chromosome 14 in artichoke, most likely during the process of domestication. The classification as acrocentric, of four chromosome pairs in cardoon and three chromosome pairs in globe artichoke was further supported by the distribution of the (GA)_10_ and (CA)_10_ SSR oligonucleotides along only one side of those chromosome arms, compared to the distribution on both arms of all the remaining chromosome pairs of the complement detected by FISH.

Chromosome characterization by FISH labelling was preceded by FISHIS on nuclei suspensions. This procedure was initially developed to label chromosomes in suspension for flow karyotyping and sorting, as an effective method to discriminate, purify and isolate specific plant chromosomes (Lucretti et al. 2014). Here we propose to use FISHIS on nuclei suspensions as a fast way for screening labelling probes. We found this method particularly effective for the selection of probes producing strong, discrete and localized hybridization signals, the kind usually most useful for identification of chromosomes; while it was less valuable for sequences more scattered and widespread.

The rDNA genes sites identified using traditional FISH analysis agrees, as number, with that reported in the recent work of Falistocco (2016), but appeared localized on acrocentric chromosomes in both crops. This ascertainment is consistent with Roa and Guerra (2012), who observed a very high frequency of rDNA sites on the short arms of acrocentric chromosomes in several genera.

The publication of the globe artichoke genome sequence (Scaglione et al. 2016) showed a high level of SSRs in artichoke DNA (41.73%) with di-nucleotides as the most frequent class (73%). Our FISH analysis with SSR oligonucleotides confirms the abundance of di-nucleotides, mainly (AG)_10_ and (AC)_10_ localized at telomeric and subtelomeric positions. These di-nucleotides co-localize in their FISH distribution and are probably organized in alternating tandem clusters, both in cultivated cardoon and globe artichoke. The (GA)_10 _and (CA)_10_ hybridization patterns enabled us to pair homologous chromosomes and to discriminate chromosomes 3, 5 and 8 which, compared to other chromosomes, had a wider distribution of SSRs on the long arms. The di-nucleotide localization at only one end of all acrocentric chromosomes enabled their identification in very condensed metaphases where the centromeric primary restriction is seldom visible. A *Cynara* specific centromeric probe would help in better defining chromosome arms in future studies.

Scaglione et al. (2016) also reported that AT di-nucleotides are quite abundant in the genome of globe artichoke, but it was not possible to detect any FISH signal with the (AT)_12_ probe for us. This may be due to a low level of AT repeats in each cluster of tandem repeats and/or to a very scattered distribution of AT in the genome. The self-complementary nature of the di-nucleotide could also contribute to reducing the amount of available probe for hybridization to chromosome DNA.

## Conclusion

Here we propose the karyo-morphological and molecular karyotype and the first ideogram of both cultivated cardoon and globe artichoke. Our results enable the identification of chromosomes pairs 3, 5 and 8 and the discrimination of acrocentric chromosomes in the complement of the two crop. Their karyotype revealed close affinity, but also chromosome structural variation among the two *Cynara cardunculus* varieties. Differences have been detected in the number of acrocentric chromosome, with cardoon showing an additional chromosome pairs, and also in the DNA content of the two varieties. The proposed karyotypes could help future anchoring of pseudomolecules from globe artichoke genome sequencing to chromosomes and contribute in locating important genes involved in the divergent evolution and domestication of *Cynara cardunculus*, for example, those associated with the development of different leaf structure and flower architecture.

## Disclaimer

Andrea Gennaro is employed with the European Food Safety Authority (EFSA), the present paper is published under the sole responsibility of the authors. The positions and opinions presented in this paper are those of the author alone and are not intended to represent the views or scientific works of EFSA.
